# *Notes from the Field:* Scrub Typhus Outbreak — Los Lagos Region, Chile, January–February 2023

**DOI:** 10.15585/mmwr.mm7227a4

**Published:** 2023-07-07

**Authors:** Thomas Weitzel, Constanza Martínez-Valdebenito, Gerardo Acosta-Jamett, Katia Abarca

**Affiliations:** ^1^Laboratorio Clínico, Clínica Alemana, Facultad de Medicina Clinica Alemana, Universidad del Desarrollo, Santiago, Chile; ^2^Instituto de Ciencias e Innovación en Medicina, Facultad de Medicina Clínica Alemana, Universidad del Desarrollo, Santiago, Chile; ^3^Departamento de Enfermedades Infecciosas e Inmunología Pediátricas, Escuela de Medicina, Pontificia Universidad Católica de Chile, Santiago, Chile; ^4^Laboratorio de Infectología y Virología Molecular, Red Salud UC-Christus, Santiago, Chile; ^5^Instituto de Medicina Preventiva Veterinaria and Center for Disease Surveillance and Evolution of Infectious Diseases, Facultad de Ciencias Veterinarias, Universidad Austral de Chile, Valdivia, Chile.

During January 14–February 14, 2023, a total of 36 cases of suspected scrub typhus were reported from the Los Lagos Region in southern Chile. Scrub typhus is a bacterial disease caused by one of three *Orientia* spp. and transmitted through bites from infected chiggers (larval mites).

## Investigation and Outcomes

Patients with reported cases fulfilled at least two of the following three criteria in the Chilean surveillance case definition: 1) acute febrile illness; 2) a generalized maculopapular rash; and 3) presence of a necrotic lesion (eschar). Blood and eschar material collected from patients with suspected scrub typhus cases were sent to the national reference laboratory in Santiago and tested using genus-specific quantitative real-time polymerase chain reaction (qPCR) testing (Orien16S), which detects all known *Orientia* species (*1*). Demographic and clinical data were collected by the treating physicians using the national surveillance system questionnaire. This study was reviewed and approved by the Pontificia Universidad Católica Institutional Review Board.^†^

In 28 (78.0%) of the 36 suspected cases, *Orientia* spp. was identified by qPCR, including 16 (57.0%) from eschar material only, one (4.0%) from the buffy coat fraction of the collected blood specimen only, and 11 (39.0%) from both sources. Twenty-two (79.0%) confirmed cases were acquired at the patients’ place of residence, and six (21.0%) were acquired during travel in the Los Lagos Region. The number of confirmed cases reported in the Los Lagos Region as of February 2023 represented an increase of nearly 450% over the mean number of cases reported during the preceding 8 years (5.1) (Figure). Most confirmed cases occurred among males (64.3%); two thirds (67.9%) occurred among adults aged 18–50 years (median age = 46 years; range = 8–71 years). The typical clinical presentation included fever (85.7%), accompanied by skin manifestations (eschar [100.0%] or maculopapular rash [89.3%]), as well as nonspecific signs and symptoms including headache (85.7%), myalgias (78.6%), chills (75.0%), and night sweats (57.1%). Among patients for whom laboratory data were available, abnormalities included elevated transaminases in 96.0% (21 of 22), C-reactive protein in 84.0% (16 of 19), thrombocytopenia in 22.0% (five of 23), and leukopenia in 30.0% (seven of 23). Twenty-six (93.0%) patients reported contact with vegetation or firewood during domestic (42.0%), occupational (30.0%), or leisure (19.0%) activities. All patients received doxycycline treatment and recovered without complications.

**FIGURE:**
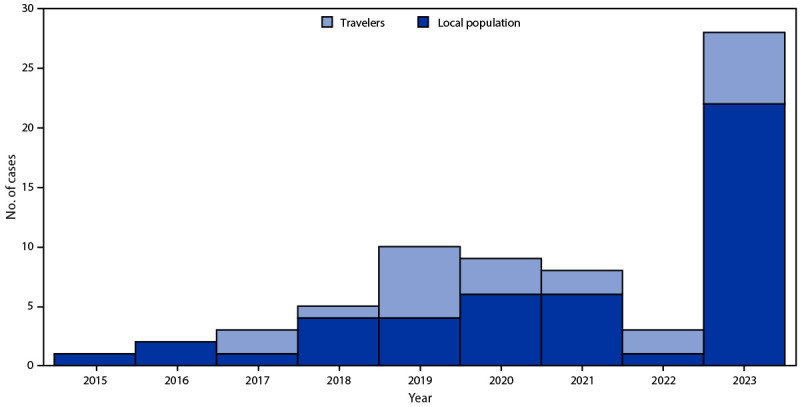
Reported scrub typhus cases by affected population — Los Lagos Region, Chile, January 14–February 14, 2015–2023

## Preliminary Conclusions

Scrub typhus is the oldest known vectorborne infection, and until recently, has almost exclusively been reported from certain regions within the Asia-Pacific region (the tsutsugamushi triangle), where it is caused by *Orientia tsutsugamushi* (*2*). In that region, approximately 1 million cases are reported each year, with a case fatality rate of approximately 7% if not adequately treated (*2*). Scrub typhus was recently discovered in southern Chile (*3*), occurring over a geographic range of almost 1,240 miles (2,000 km) from the Biobío Region in central Chile to Tierra del Fuego in the south. Molecular analyses have identified a novel *Orientia* species (*Candidatus* Orientia chiloensis) as the causative pathogen (*4*). Vector studies in the Los Lagos and Aysén regions suggested larval trombiculid mites of the genus *Herpetacarus* (commonly known in the United States as chiggers) as disease vectors (*5*).

Understanding the reasons for the observed increase in scrub typhus cases requires further eco-epidemiologic studies. Scrub typhus in Chile displays a marked seasonality, with 97% of cases to date occurring during the austral summer months of December–March (Chilean Rickettsia and Zoonosis Research Group, unpublished data, January 2023). Apart from climatic factors, the outbreak might also be related to an increase in outdoor activities after 2 years of pandemic restrictions as well as growing awareness of the disease, resulting in increased testing and reporting. Because of its nonspecific clinical characteristics, scrub typhus might be easily overlooked, and diagnosis requires a high index of suspicion. Rapid diagnosis and treatment, however, are crucial to avoid severe disease and possible complications, such as pneumonia, renal failure, and meningoencephalitis. If infection is suspected, treatment with doxycycline should be initiated without delay (*2*).

Exposure to trombiculid mites is associated with outdoor activities and affects not only residents of rural areas, but also travelers on camping and trekking trips. The growth of ecotourism in southern Chile has increased the importance of raising awareness among physicians worldwide who see ill travelers returning from the region. No vaccine is available to prevent scrub typhus; to prevent exposure to mites, travelers should avoid contact with lower vegetation and soil, wear long sleeves and pants, treat boots and clothing with the insecticide permethrin (0.5%), and use insect repellents containing DEET or other active ingredients registered by the Environmental Protection Agency for use against chiggers, on exposed skin and clothing (*2*). 

## Footnotes

† C.F.R. part 46; 21 C.F.R. part 56.
